# Methadone alters transcriptional programs associated with synapse formation in human cortical organoids

**DOI:** 10.1038/s41398-023-02397-3

**Published:** 2023-05-06

**Authors:** Ila Dwivedi, Andrew B. Caldwell, Dan Zhou, Wei Wu, Shankar Subramaniam, Gabriel G. Haddad

**Affiliations:** 1 Department of Pediatrics, School of Medicine, University of California, San Diego, La Jolla, CA USA; 2 Department of Bioengineering, University of California, San Diego, La Jolla, CA USA; 3 Department of Cellular & Molecular Medicine, School of Medicine, University of California, San Diego, La Jolla, CA USA; 4 Department of Nanoengineering, University of California, San Diego, La Jolla, CA USA; 5 Department of Computer Science & Engineering, University of California, San Diego, La Jolla, CA USA; 6 Department of Neurosciences, School of Medicine, University of California, San Diego, La Jolla, CA USA; 7 Rady Children’s Hospital, San Diego, CA USA

**Keywords:** Addiction, Molecular neuroscience, Stem cells

## Abstract

Opioid use disorder (OUD) among pregnant women has become an epidemic in the United States. Pharmacological interventions for maternal OUD most commonly involve methadone, a synthetic opioid analgesic that attenuates withdrawal symptoms and behaviors linked with drug addiction. However, evidence of methadone’s ability to readily accumulate in neural tissue, and cause long-term neurocognitive sequelae, has led to concerns regarding its effect on prenatal brain development. We utilized human cortical organoid (hCO) technology to probe how this drug impacts the earliest mechanisms of cortico-genesis. Bulk mRNA sequencing of 2-month-old hCOs chronically treated with a clinically relevant dose of 1 μM methadone for 50 days revealed a robust transcriptional response to methadone associated with functional components of the synapse, the underlying extracellular matrix (ECM), and cilia. Co-expression network and predictive protein-protein interaction analyses demonstrated that these changes occurred in concert, centered around a regulatory axis of growth factors, developmental signaling pathways, and matricellular proteins (MCPs). TGFβ1 was identified as an upstream regulator of this network and appeared as part of a highly interconnected cluster of MCPs, of which thrombospondin 1 (TSP1) was most prominently downregulated and exhibited dose-dependent reductions in protein levels. These results demonstrate that methadone exposure during early cortical development alters transcriptional programs associated with synaptogenesis, and that these changes arise by functionally modulating extra-synaptic molecular mechanisms in the ECM and cilia. Our findings provide novel insight into the molecular underpinnings of methadone’s putative effect on cognitive and behavioral development and a basis for improving interventions for maternal opioid addiction.

## Introduction

Over the past two decades, Opioid Use Disorder (OUD) among pregnant women has become an epidemic in the United States [1]. Between 2010 and 2017, there was a 131% increase in the estimated rate (per 1000 delivery hospitalizations) of maternal opioid related diagnoses, including long-term use and dependence [2]. This was accompanied by a parallel surge in the percentage of expectant mothers seeking treatments for opioid addiction [3]. The standard of care for maternal OUD is Medication-Assisted Treatment (MAT) with methadone [3, 4], a synthetic mu (μ)-opioid analgesic that minimizes deleterious opioid withdrawal symptoms and risk-taking behaviors that lead to relapse or overdose [5, 6].

Despite its utility in adults, methadone’s ability to readily enter fetal circulation and accumulate in neural tissue has led to concerns regarding its effects on brain development *in utero* [4, 7, 8]. Clinically, prenatal methadone exposure is linked with increased incidence and severity of Neonatal Abstinence Syndrome (NAS), characterized by central nervous system hyperirritability and autonomic nervous system dysfunction [9, 10]. Crucially, longitudinal studies of exposed infants [11, 12], mice [13], or rats [14–16] also revealed long-term psychomotor and cognitive sequelae including impaired learning, memory, social, and motor skills, as well as depression and anxiety.

Investigations into the cellular and molecular etiology of these cognitive and behavioral impairments point to deficits in neuronal connectivity and communication that may arise during early development. Diffusion tensor imaging studies of human neonates and adults exposed to methadone maintenance therapy uncovered microstructural changes in cerebral white matter tracts, indicating potential axonal damage [17–20]. The earliest studies of methadone in rats demonstrated that the drug diminishes neurotransmitter content, uptake, and release [21, 22] and influences synaptogenesis [23, 24]. Treatment of rat neuronal cultures with other μ-opioid receptor agonists like morphine also yielded reductions in neurite outgrowth and pre- and post-synaptic puncta densities [25]. In addition, morphine, endogenous μ-opioids, and methadone have been shown to affect central and peripheral neuronal excitability and communication [26–29].

Taken together, these studies suggest that prenatal methadone exposure may lead to long-term neurocognitive sequelae by disrupting mechanisms of neural connectivity and communication. Consequently, we hypothesized that exposure to the drug during early cortical development in humans would likely alter the fundamental molecular mechanisms underlying synaptogenesis.

Nevertheless, most of these prior studies assessing methadone’s effects on neurodevelopment have been complicated by duration of drug exposure, model organism, or subject age [30]. Investigations have also largely been conducted in murine models, whose developmental mechanisms and timelines differ significantly from humans [31, 32]. The few studies done in human subjects are postnatal and complicated by *in utero* exposure to other substances, opioid-based pharmacotherapies for NAS, length of MAT, and maternal pathophysiology. Moreover, ethical and logistical complications have led to a dearth of human fetal tissues available for research. As a result, methadone’s direct effects on human fetal brain development remain largely uncharted.

To address these limitations, we utilized human iPSC-derived three-dimensional models of cortical development called cortical organoids (hCOs) [33]. These contain multiple cell types and undergo spatial organization characteristic of the in vivo fetal cortex, eliminating postnatal factors that have confounded previous studies of prenatal methadone exposure [33–36]. By 2 months of differentiation, hCOs reproducibly consist of polarized neuroepithelium-like rosettes arranged around proliferative neural progenitors (β-catenin + ), as well as mature (NeuN/MAP2 + ) glutamatergic (VGLUT1 + ) neurons, and an emerging population of 8 to 10% glial cells (GFAP + ) [33, 37].

To study the mechanisms underlying abnormalities resulting from methadone exposure during early human brain development, we conducted bulk mRNA sequencing of hCOs chronically treated with a therapeutically relevant concentration of 1μM methadone. This concentration falls within the range (0.8 to 1.7 µM) of methadone found in maternal plasma, which significantly and positively correlates with levels detected in umbilical cord blood [7, 38–41]. The organoids were treated for a total of 50 days, throughout a period of growth and maturation leading up to the onset of synapse formation at 2-months [33, 37].

This treatment paradigm models a clinical condition of methadone maintenance treatment beginning in the first trimester of pregnancy. Coupled with high-depth bulk RNA-sequencing as well as post-hoc gene ontology, co-expression module, protein-protein interaction, upstream regulator, and protein-level analyses, this methodology enabled us to dissect how methadone alters molecular mechanisms underlying neural development in the fetal cortex. The resulting findings lay the groundwork for understanding neurocognitive deficits arising from prenatal methadone exposure and improving pharmacotherapies for maternal OUD.

## Methods and Materials

### Human iPSC culture and cortical organoid generation

Human iPSC lines, A and B, derived from two healthy adult male individuals, were used to generate hCOs for RNA-sequencing. A third human iPSC line, WT83, derived from a healthy adult male individual, was additionally used to develop hCOs for Western blot analysis. Digital karyotyping with Illumina Human Core Exome Arrays (Illumina, San Diego, CA) was used to confirm cellular identity as well as chromosome and copy number stability in culture, and testing for mycoplasma contamination was performed. iPSCs were derived from fibroblasts acquired with informed consent from all individuals and utilized in accordance with UC San Diego’s Institutional Review Board (IRB) guidelines and regulations.

iPSCs were cultured in feeder-free conditions on Matrigel (Corning, Corning, NY) coated plates, and fed daily with mTeSR1™ medium (Stem Cell Technologies, Vancouver, Canada). hCOs were then generated and maintained using methods described by Muotri and colleagues [33].

### Treatment with methadone

Methadone (Sigma-Aldrich, St. Louis, MO) was dissolved in sterile nuclease-free water (Invitrogen, Waltham, MA) and diluted to a working concentration of 1 μM in fresh stage-specific medium each day of media change. Treatment began on Day 9 of organoid culture, the first day of the neural proliferation stage, and concluded at Day 60. Nuclease-free water was used as a vehicular control.

### Cortical organoid collection and RNA isolation

Cortical organoids were collected 2 months (60 days) after initiating organoid culture. Each well of hCOs (15–20 organoids) was a separate biological replicate for a given treatment condition (i.e., treated or untreated). hCOs were aspirated in 1 mL of medium and centrifuged at 3000 g for 5 min at room temperature. The pellet was resuspended in 1 mL cold (4 °C) DPBS (Corning, Corning, NY) and centrifuged again at 4 °C and 21,000 g for 10 min. Pellets were snap frozen in 1.5 mL Eppendorf tubes using dry ice and stored at −80 °C prior to RNA extraction. RNA was extracted from frozen organoid pellets using the Direct-Zol Miniprep Plus Kit (Zymo, Irvine, CA) according to the manufacturer’s instructions.

### RNA-sequencing data generation

Total RNA was sent to the UC San Diego Institute for Genomic Medicine (IGM) for quality assessment, library preparation, and sequencing. Only samples with RNA Integrity Numbers (RIN) > 7 were selected for library preparation. PolyA+ selected libraries were prepared using the TruSeq mRNA Stranded Library Prep Kit with TruSeq UDI96 indexed adaptors (Illumina). Samples were multiplexed and sequenced on the Illumina NovaSeq 6000 S4 to produce approximately 100 million, 100 base pair, paired end reads per sample. 3 control and 3 methadone-treated samples were sequenced from cell line A, and 4 control and 4 treated samples from cell line B. No samples were excluded from the analysis, given comparable RINs > 7 and read numbers close to the 100 million read per sample target amount. RINs and total sequenced reads per sample are provided in Supplementary Table S1.

### RNA-sequencing differential expression analysis

Raw fastq file quality assessment and read alignment to the hg19 genome (GRCh37, RefSeq GCF_000001405.13) [42] were performed through the *FastQC* (v1.0.0) [43] and RNA-Seq Alignment (*STAR*, v2.0.2) [44] applications, respectively, in the Illumina BaseSpace Sequence Hub. Mapped reads were assigned to genomic meta-features (genes) using the *Rsubread* (v2.6.4) [45] function *featureCounts* in R. Expression level filtering was performed using the *edgeR* (v3.34.1) [46] function *filterbyExpr*. TMM normalization factor [47] calculations were also conducted using *edgeR*. Mean-variance trends and gene-specific weights were determined via the *voom* function in the R package *limma* (v3.48.3) [48, 49]. Normal distribution of log CPM expression values by *voom* also accounted for the natural heteroscedasticity of count probability distributions. Differential expression analysis was then conducted by fitting the *voom* output to a linear model using the *lmFit* function in *limma*. Cell line and treatment condition were incorporated as covariates while contrasting methadone treated versus untreated control samples. Genes were ranked in order of evidence for differential expression using the empirical Bayes (eBayes, *t*-value) method (*n* = 17,651). Significantly differentially expressed genes (DEGs) were selected based on the confident effect size of their log_2_(Fold Change) values at FDR < 0.05. This was represented by a “confect score” calculated by *TopConfects* (v1.8.0) [50] in R. Genes with |Confect Score | ≥ log_2_(1.5) were considered DEGs.

### Gene overlap testing

The Fisher exact-test p-value and odds ratio were used to test the significance of overlap and strength of association, respectively, between the DEGs from cell lines A and B. These were calculated via the R package *GeneOverlap* [51]. Rank-rank hypergeometric overlap analysis (RRHO) and heatmap construction were done with the *RRHO2* [52, 53] package in R.

### Gene ontology and gene set enrichment analysis

To identify transcriptional signatures associated with specific cellular components, gene set enrichment analysis (GSEA) [54] was performed using the *fGSEA* (v1.18.0) [55] package in R with the Gene Ontology Cellular Component (GO-CC) database [56, 57] as a reference as well as all eBayes ranked genes and associated *limma* t-statistic values as input. The top 20 terms with the lowest FDR-adjusted p-values were selected and sorted by their normalized enrichment scores (NES).

To determine which molecular functions the DEGs associated with each top cellular component were enriched for, we applied a hypergeometric test using the GO-Molecular Function (GO-MF) database in the *GOrilla* web application [58], setting eBayes ranked genes as background (*p* < 10^−3^). Resulting GO-MF terms were arranged hierarchically and non-redundantly by semantic similarity via the *REVIGO* web-application [59]. *REVIGO* significance values and term hierarchy were used to visualize GO-MF enrichment as a circle plot using the *CirGO* package [60] in *Python* (v3.9.12) (Data File S1).

The molecular role of each synaptic DEG was determined based on its association with enriched GO Molecular Function categories and through manual searches using the *OMIM* [61, 62]*, GeneCards* [63, 64], and *NCBI Gene* [65] databases. DEGs were grouped into categories based on the representative terms found in the *CirGO* output. ECM-associated DEGs were categorized by their overlap with Core- or Matrisome-Associated genes in *MatrisomeDB* [66]. Genes in each category were then grouped according to descriptions provided in reviews by Naba and colleagues [67–69], who generated *MatrisomeDB*. Major collagen [70] and proteoglycan types [71, 72] were identified based on descriptions in the literature. As before, *OMIM, GeneCards*, and *NCBI Gene* databases helped categorize genes not identified in these sources (Data File S2).

### Modular gene co-expression and protein-protein interaction network analysis

Co-expression modules were identified through the Bioconductor *cemitool* package [73] in R, using eBayes ranked genes as input with the parameters apply_vst=FALSE and directed=TRUE. Counts were corrected for cell line differences using the *removebatcheffects* function and transformed using the *voomwithqualityweights* function in *limma*. GO-CC *fGSEA* was performed on each module, and DEGs from the top two modules with the greatest similarity in cellular component enrichment were combined for network analysis.

Edges between DEGs from the first two modules were acquired from *STRINGDB* (v.11.0) [74] and filtered to include only physical interactions (medium threshold = 0.7). Network edges and nodes were imported into *Cytoscape* (v3.9.1) [75], and top hub genes ranked by EcCentricity score [76] were identified using *CytoHubba* [77]. *MCODE* [78] determined highly interconnected clusters of proteins in each network via default network scoring and cluster finding parameters.

### Upstream regulator analysis

Ingenuity Pathway Analysis (*IPA*; Qiagen Inc., Hilden, Germany) [79] was used to identify upstream regulators of synaptic, ciliary, and ECM DEGs from the top two co-expression modules. Each regulator was assigned a p-value representing the overlap between DEGs and known targets, and a z-score to infer regulator activation states. Endogenous molecules (excluding exogenous toxicants, drugs, and reagents) were ranked in order of significance (Data File S3).

### Western blot analysis

3-month-old hCOs derived from cell lines A and WT83 were treated with 0, 1, or 10 µM methadone for 4 weeks, then isolated for protein extraction at 4-months. Each well of hCOs (25–30 organoids) was considered a biological replicate, with at least 3 replicates tested per condition across both cell lines. Replicates were collected from four experiments, each with batch-matched control and treatment conditions. Whole brain tissue from a healthy, adult mouse was used as a positive control.

Samples were lysed in 10X RIPA buffer containing protease and phosphatase inhibitors and homogenized with a glass-teflon homogenizer (Thomas Scientific, Swedesboro, NJ). Homogenates were centrifuged for 10 min at 10,000 *g* and 4 °C, and protein concentration was determined using a Bio-Rad Protein Assay Kit (Bio-Rad, Hercules, CA). 30 µg of total protein was separated on a NuPAGE 4–12% Novex Bis-Tris gel (ThermoFisher, Waltham, MA) and transferred to polyvinylidene difluoride membrane (Millipore, Burlington, MA). Membranes were blocked for 1 h and incubated in PBST with 5% BSA containing primary antibodies overnight at 4 °C on a shaker. The primary antibodies used were rabbit anti-Thrombospondin 1 (37879, Cell Signaling Technology, Waltham, MA; 1:500) and rabbit anti-GAPDH (PA1-987, ThermoFisher; 1:1000). Membranes were then incubated with the secondary antibody Goat anti-Rabbit IgG (H + L) HRP (A32731, Invitrogen; 1:2000) for 1 h at room temperature and developed using an ECL Kit (ThermoFisher). Immunoreactive bands were visualized using the Bio-Rad ChemiDoc XRS with enhanced chemiluminescence (Perkin-Elmer, Waltham, MA), and relative band intensity was analyzed using the ImageLab software (Version 3.0, Bio-Rad).

### Statistical analyses

Statistical details specific to the programs or functions used for analysis are provided in the Methods, Results, and Figure Legends. Unless otherwise specified, bioinformatic analyses were conducted using *R* (v4.1.3) [80] in *RStudio* (v1.4.1717) [81].

Total sample numbers per condition for RNA-sequencing were established based on prior estimates of biological replicates required to appropriately power differential expression analyses using *limma* and similar programs [48, 82–84]. Multi-dimensional scaling of *voom* calculated gene expression values was performed using the *glMDSplot* function in the R package *Glimma* (v2.2.0) [85]. The resulting dimensions (covariates) were sorted in order of decreasing variance along them. Variability in sample-level gene expression was represented in the form of z-scores plotted in heatmaps using the *heatmap.2* function from *gplots* (v.3.1.3) [86].

Western blot relative band intensity values were statistically analyzed in GraphPad Prism (GraphPad Software, La Jolla, CA), using a one-way *ANOVA* comparing each treatment group to the control group. Significance was defined as *p* < 0.01 (**).

### Data visualization

**Fig. 1:**
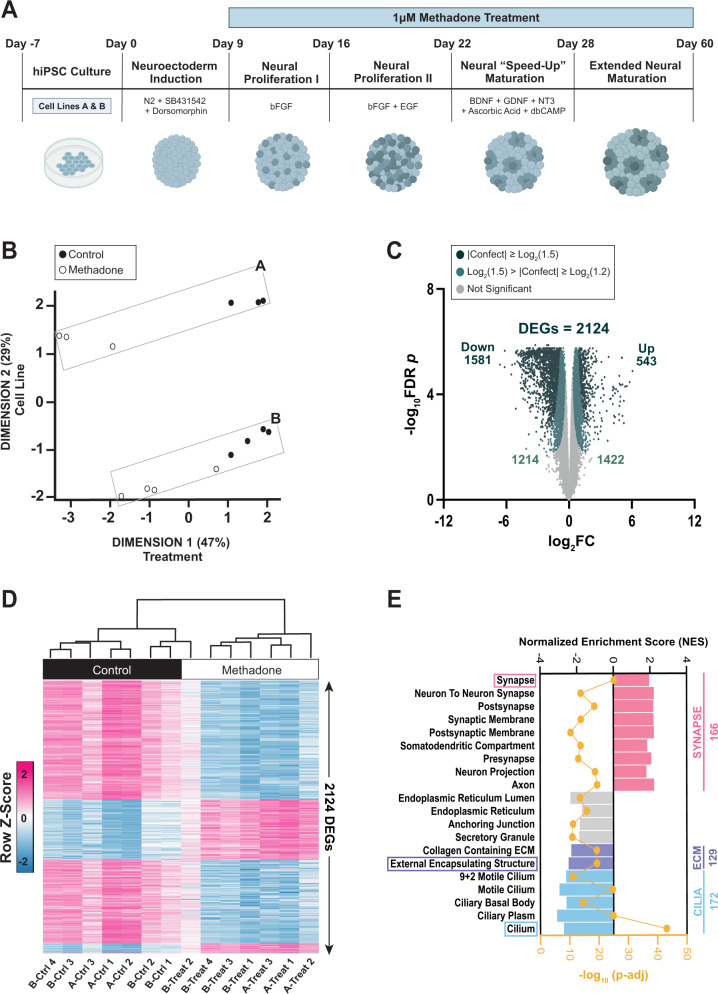
Methadone elicits a robust transcriptional response in 2-month-old hCOs. **A** Timeline of cortical organoid generation and methadone treatment. (**B**) Multi-dimensional scaling of TMM-normalized expression data and metadata from control and methadone-treated hCO samples derived from cell lines **A** & **B**. **C** RNA-seq volcano plot of all (eBayes ranked) genes, distinguished by their confident effect size ‘confect’ score cutoffs. Genes with |Confect | ≥ log_2_(1.5) and FDR < 0.05 were considered DEGs (DEGs = 2124), after adjusting for cell-line differences. For each gene, log_2_ (Fold Change) effect size values are shown on the x-axis and Benjamini-Hochberg adjusted absolute log_10_ *p*-values are along the y-axis. **D** Heat map depicting the sample-level expression (z-scores) of the 2124 DEGs across both cell lines. **E** Top 20 *fgsea* enriched GO Cellular Component gene sets based on absolute log_10_ FDR-adjusted p-values, ranked according to the direction (positive or negative) of their normalized enrichment scores (NES) and grouped by cellular component. Values to the right of the graph indicate the number of DEGs associated with each of the top 3 enriched GO-CC gene sets.

**Fig. 2:**
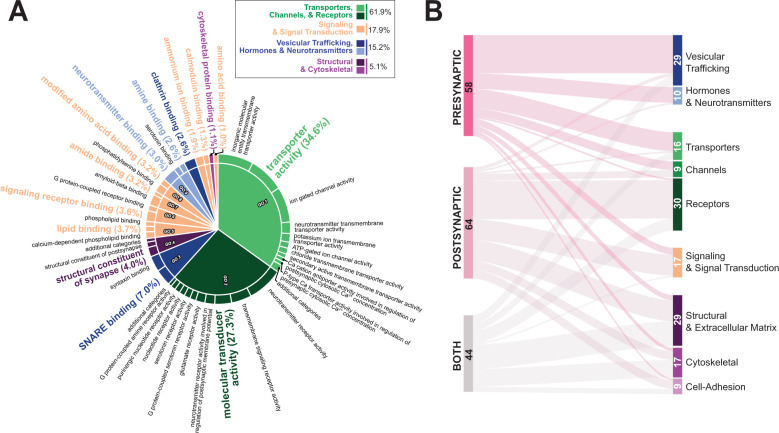
Methadone alters the expression of pre- and post-synaptic functional constituents. **A** Molecular function ontology of synapse-specific DEGs, determined via a hypergeometric test using a background of eBayes-ranked expressed genes and a GO gene set significance threshold of *p* < 10^−3^. “Parent” or representative gene sets (molecular function categories) are presented in the inner circle and emphasized with large, colored font. Semantically similar descendants of these parents are represented in black font. Terms in each category are sorted in descending order by absolute log_10_ *p*-values. Percentages indicate the relative membership of each parent gene set based on the number and significance of the constituent descendant terms. Gene sets were broadly summarized and colored according to the categories shown in the boxed insert. **B** Molecular functions of 166 synapse-associated DEGs. Node values and sizes, as well as edge thicknesses, represent the number of DEGs belonging to each GO cellular component category (pre-synapse, post-synapse, both) or molecular function.

**Fig. 3:**
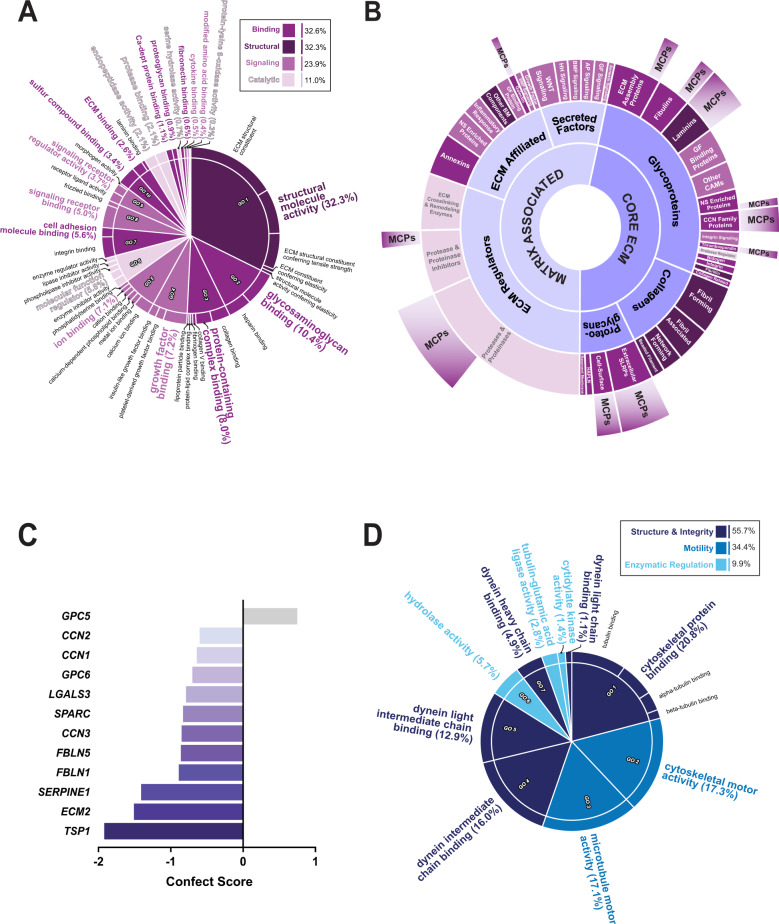
Methadone induces transcriptional changes associated with ECM and ciliary structure and function. **A** Enriched molecular function ontology of ECM-specific DEGs compared to a background of eBayes-ranked expressed genes. Parent and descendant terms are determined and visualized as described in Fig. 2A. Broad categories of enriched ECM gene sets are summarized in the boxed insert. **B** Categorization of ECM-associated DEGs. The inner two circles group molecules according to their designations in *MatrisomeDB*. The third circle organizes molecules in each Matrisome category based on their functions. The outermost bars indicate the number of matricellular proteins (MCPs) in each category. Segment sizes correspond to the number of DEGs in each category. **C** All MCPs classically identified in the brain that were differentially expressed in response to methadone. Genes are ranked by their confect score values. **D** Enriched molecular function ontology of ciliary-DEGs compared to a background of eBayes-ranked expressed genes. ECM, extracellular matrix, CAM Cell adhesion molecule, GF Growth factor, NS Nervous system, SLRP Small-leucine rich proteoglycan, HAPLN Hyaluronan and proteoglycan link, BM Basement membrane, HH Hedgehog, AP Angiopoetin.

**Fig. 4:**
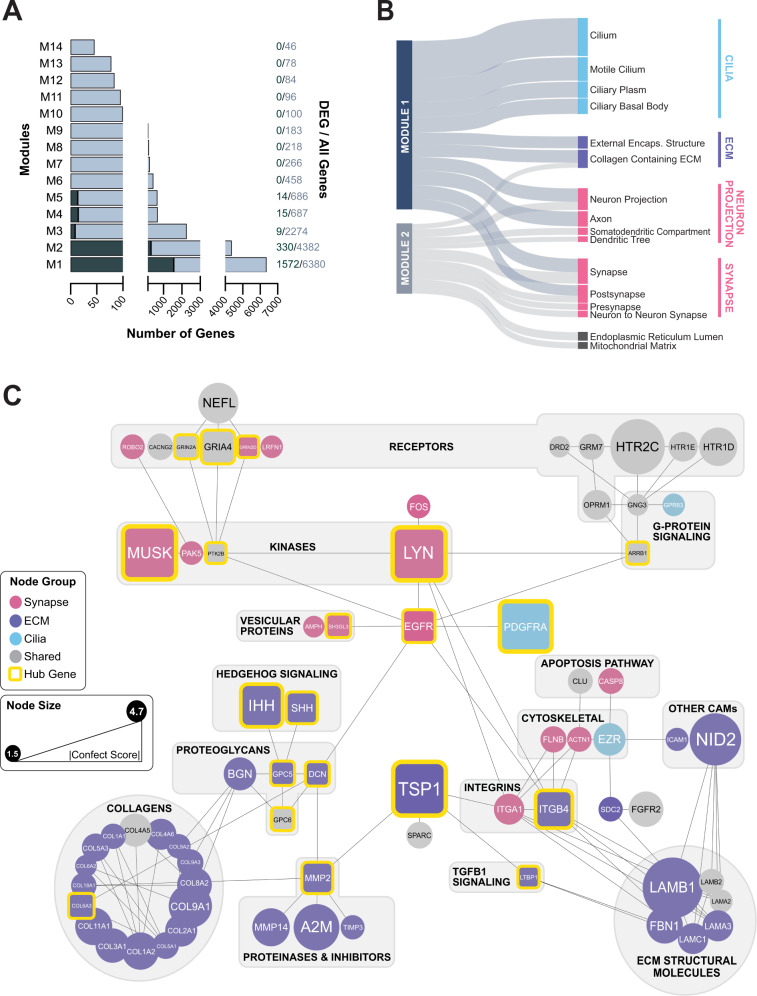
Co-expressed synaptic, ECM, and ciliary genes encode physically interacting proteins. **A** Sizes of all co-expression modules identified among all eBayes-ranked genes following correction for cell line differences. The number of DEGs in each module are shown in green. Module sizes and DEG membership are indicated as ratios to the right of each bar. **B** Top enriched GO-CC terms associated with modules M1 and M2 based on absolute log_10_ *p*-values, which are represented by the relative thickness of the nodes and edges in the plot. **C** Protein-protein interaction network of synapse (pink), ECM (purple), and cilia (blue) associated genes belonging to modules M1 and M2. ‘Neuronal Projection’ genes were grouped with synaptic genes based on semantic similarity. Nodes in grey are proteins belonging to more than one cellular component category. Node sizes reflect the relative magnitude of absolute confect scores, while edges indicate predicted physical interactions in the brain according to *STRINGDB*. The top hub genes identified by *CytoHubba* using the EcCentricity metric are emphasized in square boxes with yellow borders. Major functional groups are highlighted using labeled grey boxes.

**Fig. 5:**
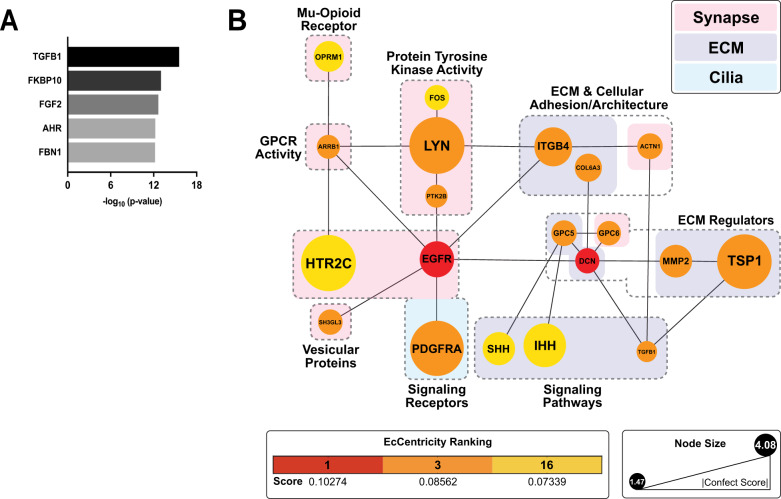
TGFβ1 is a key upstream regulator of synaptic and extra-synaptic DEGs. **A** Top 5 endogenous protein upstream regulators of the synaptic, ECM, and ciliary genes belonging to modules M1 & M2 in Fig. 4B, identified via *IPA*. **B** Predicted physical interactions between the top 20 hub genes in a synaptic-extra-synaptic network including the upstream regulator TGFβ1. Node colors represent EcCentricity centrality rankings, while node sizes reflect the relative magnitudes of absolute confect scores. Genes are clustered according to their roles in each cellular component.

**Fig. 6:**
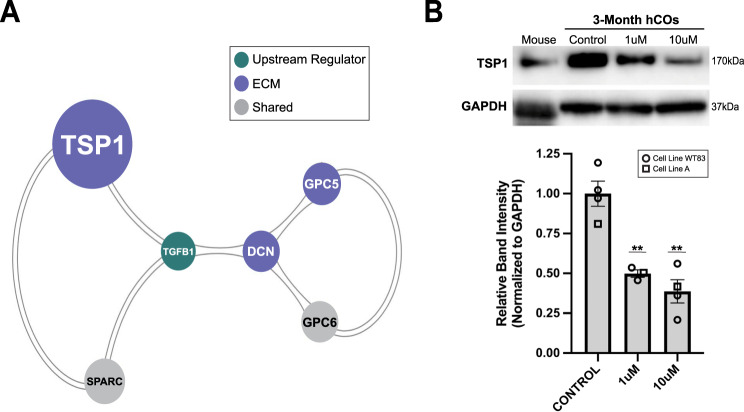
Methadone alters a highly interconnected growth factor-MCP regulatory axis. **A** A highly interconnected network of MCPs and the upstream regulator TGFβ1, identified through MCODE (Cluster score = 2.8, node score cutoff = 0.2). **B** Western blot of TSP1 levels in mouse brain tissue and WT83 cell line derived 4-month-old hCOs, which had been treated with 0 (Control), 1, and 10 µM methadone for 4 weeks. The Densitometry histogram depicts the expression level of TSP1 normalized to GAPDH in 4-month-old hCOs derived from cell lines A and WT83. Expression levels are represented by relative Western blot band intensities, and bars represent mean band intensities for each condition ± SEM. One-way ANOVA followed by a post-hoc Bonferroni test was used to determine significance of change in each treatment condition compared to the untreated control hCO samples (*F*_1µM_ = 27.52, *F*_10µM_ = 32.55, **p < 0.01).

hCO schematics were created with BioRender (https://biorender.com/) (Fig. 1A). Bar, bar/line, and volcano plots were generated in GraphPad Prism (Figs. 1C, 1E, 3C, 4A, 5A, 6B and S1A, S2A, B). Sankey plots were made with the SankeyMATIC tool (https://sankeymatic.com/) (Figs. 2B and 4B). The *MatrisomeDB* concentric circle plot was constructed in Microsoft Excel (Fig. 3B). All other methods of data representation (*CirGO* plots, networks, etc) are described above.

## Results

### Chronic methadone induces a robust transcriptional response in 2-month-old hCOs

Two-month-old hCOs generated from two iPSC cell lines, A and B, exhibited robust transcriptional responses to 50 days of chronic treatment with 1μM methadone (Fig. 1A). 4165 DEGs were detected in line A, while 1018 DEGs were identified in line B ( | Confect | ≥ Log_2_(1.5), FDR < 0.05) (Fig. S1A).

Despite differences in the magnitude of response to methadone between cell lines, multi-dimensional scaling revealed that methadone treatment was the primary source of sample-level variation (Fig. 1B). Separation of samples along the second dimension demonstrated variation introduced by baseline transcriptional differences between iPSC lines. This variability between iPSC lines has been well described in previous studies and is primarily attributed to the distinct genetic backgrounds of donors and/or mutations acquired during somatic cell reprogramming for iPSC generation or clonal expansion [87, 88].

Importantly, in both cell lines, control samples were differentiated from treated samples by unsupervised clustering according to their expression profiles, signaling a consistent response to drug treatment (Fig. S1B). Moreover, the intersection of 777 genes between cell lines A and B was statistically significant (Fisher’s exact *p* = 4.7 × 10^−272^) and the association between both lists was strong (Fisher’s odds ratio = 11.3) (Fig. S1C). The most significant overlap occurred between downregulated DEGs, which constituted most of the transcriptional response (Fig. S1D).

Taken together, this information enabled us to incorporate cell line as a covariate into the differential expression linear model and account for baseline transcriptional differences between individuals. Through this methodology we obtained 2124 DEGs whose expression was altered due to methadone treatment alone (Fig. 1C). Almost all samples across both cell lines clustered according to treatment condition based on their expression of these DEGs (Fig. 1D). Although sample B-Treat 2 clustered with controls, its high RNA quality and read counts precluded its exclusion from further analyses, enabling us to retain heterogeneity of response to methadone in our dataset (Fig. 1D; Table S1).

### Methadone alters the expression of pre- and post-synaptic functional components

Since we had hypothesized that methadone would affect synapse formation, we began by determining how the observed transcriptional response was associated with the cellular anatomy of our organoid system. Pre-ranked gene set enrichment analysis (GSEA) using the Gene Ontology Cellular Component (GO-CC) database revealed the significant enrichment of gene sets associated with neuronal synapses (Fig. 1E). We noted sets linked to both the pre- and post-synapse, distinguished by terms such as “Axon” or “Somatodendritic Compartment”, respectively. All sets had positive normalized enrichment scores (NES), trending towards transcriptional upregulation in the synapse following methadone exposure.

To identify which aspects of synaptic biology were specifically affected by methadone, we performed a ranked GO-Molecular Function (GO-MF) enrichment analysis of the synaptic DEGs and summarized the resulting non-redundant ontology terms hierarchically (Fig. 2A; Data File S1). We observed alterations in all aspects of synaptic biology, including the pre-synaptic trafficking, release and synthesis of hormones and neurotransmitters, the postsynaptic reception and response to these signaling molecules, and intracellular cytoskeletal scaffolding [89–91].

There were 166 synapse-associated DEGs, of which 58 were identified as pre-synaptic, 64 as post-synaptic, and 44 as linked to both terminals (Fig. 2B; Data File S2). To further resolve the identities of these genes, we categorized them functionally according to the GO-MF, *OMIM, GeneCards*, and *NCBI Gene* databases. Most pre-synaptic DEGs were involved with vesicular trafficking, while post-synaptic DEGs were primarily receptors or signal transduction molecules. The 44 DEGs associated with both terminals were primarily structural, involved with cytoskeletal integrity, cell-cell adhesion, or extracellular matrix (ECM) composition.

### Methadone induces transcriptional changes in an ECM regulatory hierarchy

Consistent with the identities of the 44 shared pre- and post-synaptic DEGs, we detected two GO-CC gene sets with negative NES values associated with the ECM (“external encapsulating structure” and “collagen containing ECM”) (Fig. 1E). The ECM is a critical component of the tetrapartite synapse, which otherwise consists of the presynaptic bouton, postsynaptic terminal, and supporting glial cells [92, 93]. Given the ECM’s role in synapse formation and maintenance, we studied how its composition and function had been affected by methadone treatment. To this end, we conducted a ranked GO-MF enrichment analysis of the 129 ECM-associated DEGs and summarized the resulting non-redundant terms hierarchically. Four distinct functional categories of matrix structural, binding, catalytic, or signaling molecules were enriched, encompassing the ECM regulatory hierarchy (Fig. 3A; Data File S1) [94, 95].

To parse these DEGs further, we sorted them according to *MatrisomeDB*, a collection of genes defined as core (collagens, proteoglycans, and other glycoproteins) or associated (ECM regulators, secreted factors, or other affiliated molecules) matrix proteins [66–69]. Methadone treatment disrupted the expression of genes belonging to each of these designations (Fig. 3B; Table S2; Data File S2). Glycoproteins (other than proteoglycans) accounted for over half of the core matrix proteins in our dataset (55.6%, *n* = 35/63). These included structural molecules like laminins, cell adhesion molecules, and non-structural matrix modulators belonging to the CCN or thrombospondin family of proteins [94, 95]. ECM regulators also constituted almost half (*n* = 31/66, 47%) of the matrix-associated (non-core) proteins. These were primarily matrix proteases and their inhibitors (*n* = 23/31, 74%), including a family of matrix metalloproteinases (MMPs or ADAMTS) that modulate ECM composition by hydrolyzing its components [96–98].

Notably, over a quarter of all ECM DEGs encoded known and proposed matricellular proteins (MCPs) (*n* = 37/129, 28.7%) (Fig. 3B; Data File S2) [99–105]. MCPs are non-structural proteins that modulate ECM composition and integrity through interactions with structural proteins, proteases, cell surface receptors, and growth factors [100–105]. In the developing brain, MCPs regulate mechanisms of cellular maturation, proliferation, migration, axonal guidance, and synapse formation. Among the MCP families classically found in the brain, thrombospondins (thrombospondin1, *TSP1*), *SPARC* (including *Hevin/SC1/ECM2*), the Cellular Communication Network Factors (*CCN1-3* or *CYR61*, *CTGF*, and *NOV*), glypicans (*GPC5* and *6*), galectins (*LGALS3*), plasminogen activator inhibitor (*SERPINE1/PAI-1*), and fibulins (*FBLN1* and *5*) were all differentially expressed due to methadone (Fig. 3C; Data File S2) [106–110]. Of these, *TSP1* exhibited the greatest magnitude of change in response to treatment (Fig. 3C; confect score = −1.92, FDR < 0.05). Altogether, these changes indicated that methadone alters crucial components of the ECM regulatory hierarchy that are necessary for developmental synapse formation.

### Methadone disrupts the expression of genes involved in ciliary integrity

Unexpectedly, changes to the ECM were also accompanied by the enrichment of cilium-associated gene sets (Fig. 1E). As with the ECM, the 172 ciliary DEGs were mostly downregulated, indicated by the negative NES values. These DEGs could be separated into three groups encoding motor proteins (e.g., kinesins and dyneins), cytoskeletal proteins or cytoskeleton binding proteins contributing to projection integrity, and enzymes regulating cytoskeletal or motor protein polymerization (Fig. 3D; Data File S1) [111–113]. This data revealed a concurrent, directional response to methadone by transcriptional programs informing ECM and ciliary structure and function. The overall pattern of synaptic, ECM, and ciliary enrichment was also observed in cell lines A and B individually, reinforcing a consistent and parallel change in synaptic and extra-synaptic biology in response to drug treatment (Figure S2A–C).

### Synaptic, ECM, and ciliary DEGs are highly co-expressed and encode proteins that physically interact

To investigate the relationship between DEGs belonging to the synaptic, ciliary, and ECM compartments, we performed comprehensive modular co-expression analysis of eBayes ranked genes, which yielded 14 modules of highly co-expressed genes. Of these, the top two modules retained the greatest DEG membership ratios (module M1 = 1572/6380 and module M2 = 330/4382) (Fig. 4A). GO-CC GSEA revealed that M1 and M2 were significantly enriched for gene sets associated with the synapse, ECM, and cilia (Fig. 4B, Table S3). This ontological overlap between M1 and M2 allowed us to use their intersecting DEGs for further analyses and indicated that changes to the synapse were not occurring in isolation, but in concert with changes to the ECM and cilia.

Between the first two co-expression modules, 71 proteins encoded by synaptic, ciliary, and ECM DEGs were predicted to physically interact in the brain based on experimental evidence found in *STRINGDB* (Fig. 4C). Topological analysis of this network using the node centrality index EcCentricity in *CytoHubba* identified 20 major hubs spanning each cellular component (Fig. 4C; Fig. S3). Node eccentricity reflects how easily a protein can be functionally reached by other proteins in a regulatory network, indicating its centrality of influence [76]. The MCP TSP1 appeared as a major hub based on its magnitude of change, bridging interactions between matrix proteases, structural constituents, and cell-adhesion proteins. Likewise, the epidermal growth factor receptor EGFR was central to interactions between several functional ECM and synaptic proteins. The latter included synaptic receptors like the μ-opioid receptor (OPRM1) as well as dopaminergic, serotonergic, and glutamatergic NMDA and AMPA receptors (GRIN2A, GRIN2D, and GRIA4), which were among the top 20 hubs. EGFR’s relationship with these receptors was mediated by its association with kinases like LYN, PAK5, and PTK2B and G-protein signaling molecules. EGFR was also shown to interact with the platelet-derived growth factor receptor α (PDGFRA), which is activated in primary cilia and is required broadly during CNS development [114, 115].

### TGFβ1 is a key regulator of the synaptic, ECM, and ciliary protein interaction network

We next sought to understand methadone’s influence on the regulatory hierarchy of this interaction network of co-expressed synaptic and extra-synaptic DEGs. *IPA* Upstream Regulator Analysis identified TGFβ1 as a principal regulator of the synaptic, ciliary, and ECM DEGs in the first two co-expression modules M1 and M2 (Fig. 5A; Data File S3). Upon inclusion into the interaction network described in Fig. 4C, TGFβ1 was identified as a hub based on its EcCentricity Score (Fig. S4). The top 20 DEGs with the greatest scores comprised a highly interconnected nexus of synaptic, ciliary, and ECM regulatory molecules (Fig. 5B). Via interaction with G-proteins and protein tyrosine kinases, OPRM1 linked to a cascade of signaling pathways regulated by the ECM and cilia such as PDGFRA, Hedgehog, and TGFβ1, that were physically linked to both pre-synaptic vesicular trafficking (SH3GL3) and post-synaptic neurotransmitter reception (HTR2C). EGFR and TSP1 were once again identified as major hub genes by centrality and degree of differential expression.

### TGFβ1-MCP interactions are central to the synaptic and extra-synaptic response to methadone

MCODE cluster analysis of the interaction network including TGFβ1 (Fig. S4) placed this growth factor within a tightly interconnected cluster of MCPs (TSP1, GPC5, GPC6, and SPARC) (Fig. 6A; Table S4). This finding highlighted the functional relationship between MCPs and TGFβ1 during synaptogenesis in the hCOs and emphasized their regulatory centrality in the response to chronic methadone treatment.

To validate methadone’s functional effect on this cluster, we quantified protein-levels of the most highly affected MCP, TSP1, in response to treatment (Fig. 6B). 3-month-old organoids derived from cell line A and an additional cell line WT83 were exposed to 0, 1, or 10 µM of methadone for 4 weeks and collected for protein extraction at 4-months, by which point functional synapses have been established and allowed to mature [33, 37]. TSP1 levels were significantly diminished in a dose-dependent manner by methadone treatment. These results reinforced methadone’s consistent effect on the ECM regulatory hierarchy across cell lines and demonstrated that the drug reduces functional TSP1 levels during a period of synapse formation and maturation.

## Discussion

In this study, we investigated the transcriptional effect of chronic methadone treatment on the earliest stages of cortico-genesis modeled in human iPSC-derived cortical organoids. hCOs were exposed to 1 µM methadone, a concentration that falls within the 0.8–1.7 µM range typically reported in the plasma of pregnant women [7, 38–41]. Studies support extensive materno-fetal transfer of methadone, with evidence from both humans and rats indicating accumulation of the drug in fetal tissues and cord blood throughout gestation [4, 7, 8]. At dosages required to reach withdrawal relief (100–180 mg) during pregnancy, trough concentrations of methadone in cord blood are predicted to reach the maternal reference range used to establish our treatment paradigm [4, 8]. Therefore, exposing hCOs to this concentration for 50 days beginning just after neural induction, allowed us to assess the direct effect of methadone on neural growth and maturation.

Through this methodology, our findings provided us with sufficient evidence to submit the following: first, the previously observed reductions in neural connectivity linked with methadone arise from disruptions in gene expression programs associated with synapse formation; second, these synaptogenic changes are brought about by perturbations in developmental signaling pathways originating in the ECM and cilia.

In line with prior evidence of μ-opioid-induced alterations in neural connectivity and communication [17–29], we observed significant changes in the expression of genes associated with the pre- and post-synaptic biology of chemical synapses, including the release, reception, or transduction of signals at the synapse (Fig. 2). This was congruent with the results of previous multi-electrode array (MEA) and whole-cell patch clamp studies conducted by our lab, which demonstrated dose-dependent attenuations in action potential firing and synaptic transmission (i.e., the frequency and amplitude of spontaneous excitatory post-synaptic currents) in response to methadone in 2 to 4-month hCOs [37, 116]. In conjunction with these data, our findings suggest that the methadone-induced suppression of neural network activity arises from perturbations in early pre- and post-synaptic molecular apparatuses that facilitate the establishment of synaptic transmission during prenatal development [89, 90]. Unexpectedly, synaptic gene set NES values trended towards transcriptional upregulation in response to methadone (Fig. 1E). Given the predicted physical interactions between synaptic, matrix, and ciliary DEGs, we posit that the upregulation of synaptic genes may be a compensatory response to the suppression of essential regulatory mechanisms modulated by the ECM and cilia.

The ECM’s potential role in bringing about changes at the synapse downstream of methadone is reinforced by our finding that the drug alters the expression of a vast matrix regulatory hierarchy (Fig. 3A–C, Data File S2). These results are consistent with evidence that ECM remodeling mediates opioid-induced synaptic abnormalities and may underlie drug-seeking or relapse behaviors associated with OUD [117–119]. Pivotal to the ECM hierarchy central to these changes are MCPs, modulators of cell-matrix interactions that are dynamically expressed at high levels during brain development [99, 106–110]. We identified 37 differentially expressed MCPs across several categories of core and matrix-associated proteins (Fig. 3B, C, Data File S2). Through their known involvement in growth factor signaling, these molecules mediate a balance between ECM structural proteins and the proteases that degrade them, which has downstream effects on cellular adhesion, proliferation, migration, and, ultimately, synapse formation [99, 106–110]. Several gene groups belonging to the MCP interactome were also downregulated in response to methadone treatment in our hCOs, including integrins, growth factors and their signaling molecules, collagens, and MMPs [97, 100–104].

Of the MCPs classically found in the developing brain, thrombospondin-1 (TSP1) exhibited the greatest change in expression in response to treatment and appeared as a central hub in the co-expression network of synaptic and extra-synaptic DEGs (Figs. 3C and 4C). TSP1 is a multidomain, multimeric glycoprotein that is secreted into the ECM of synapses. Its domain-specific interactions with growth factors, proteases, and cell surface receptors have proven necessary for the establishment of synaptic architecture and synaptic refinement [120–123]. Disruptions in the TSP1-TGFβ1-EGFR axis have also been implicated in reductions of synapse density in rat neuronal cultures following partial μ-opioid receptor activation by morphine [25]. Our study identified TGFβ1 as an upstream regulator of the synaptic-extra-synaptic network and both TSP1 and EGFR as a major-hubs in this network based on their degree of change and centrality (Fig. 5). Indeed, TSP1 protein levels were dose-dependently reduced after 4-weeks of treatment at later stages of hCO development, indicating that shorter-term exposure to methadone can also diminish the amount of functional TSP1 available for the proper establishment and maturation of synapses (Fig. 6C).

Alongside TSP1 and the upstream regulator TGFβ1, PDGFRA and SHH/IHH also appeared as major hubs in the synaptic-extra-synaptic regulatory network affected by methadone. PDGFRA is a receptor tyrosine kinase that localizes to primary cilia, where it mediates signals for directional cell migration and chemotaxis [114, 115]. In their capacity as sensory projections, primary cilia also contain receptors for Hedgehog, Wnt, Notch, other potent growth factors, integrins, and cadherins [111–113]. Localization of the soluble SHH and IHH ligands to primary cilia, in particular, has proven crucial for their signaling [115], which plays a prominent functional role during synapse formation and circuit assembly. Recent studies have indicated extensive crosstalk between cilia and the ECM, with ciliopathies leading to the dysregulation of ECM proteins like collagens, laminins, MMPs, and the TGFβ signaling pathway, all of which we observed in our study [124, 125].

This is the first time such transcriptional changes have been described for methadone, a full μ-opioid receptor agonist, in a human-specific model following a treatment regimen clinically relevant to maternal OUD. In this study, the use of bulk RNA-sequencing enabled us to probe molecular pathways affected by methadone with greater depth and resolution compared to available single-cell techniques, which are limited by substantial noise, inter-sample variability, fewer expressed genes per cell type, and low replicates [126]. This technique led us to identify several molecular targets, including TSP1 and TGFβ1, which we are now investigating as regulators of the cortical response to methadone and as avenues for the amelioration of sequelae arising from disruptions in synapse formation caused by the drug. Altogether, we believe that our data contribute to a mechanistic understanding of the neurobehavioral deficits associated with prenatal methadone exposure and provide a foundation upon which to improve pharmacological interventions for OUD in pregnant women.

## Supplementary information

Supplementary Figures S1-4, Tables S1-4, and Data File S1-3 Summary

Supplementary Data File S1

Supplementary Data File S2

Supplementary Data File S3

## Data Availability

All data and information required to generate, evaluate, and interpret the findings presented in this paper are provided in the methodology, main figures and supplementary materials, which will be available on the *Translational Psychiatry* website. Code used to generate the results followed the documentation for each referenced program, and all relevant parameters are detailed in the Methods. The RNA-sequencing dataset presented in this work is available at the NCBI GEO under the SuperSeries accession GSE210682. Further requests for data and inquiries may be directed to the corresponding author.
