# Supplementary material for: Methadone alters transcriptional programs associated with synapse formation in human cortical organoids
Source: Transl Psychiatry. 2023 May 6;13:151. doi: 10.1038/s41398-023-02397-3 (PMC10163238; doi:10.1038/s41398-023-02397-3)
Supplement: Supplementary file 1 — Supplementary Figures S1-4, Tables S1-4, and Data File S1-3 Summary [file 41398_2023_2397_MOESM1_ESM.pdf]

SUPPLEMENTARY MATERIALS

Methadone alters transcriptional programs associated with synapse formation in human cortical organoids

Ila Dwivedi, MS[1], Andrew B. Caldwell, PhD[2†], Dan Zhou, PhD[1†], Wei Wu, PhD[1]
Shankar Subramaniam, PhD[2,3,4,5], Gabriel G. Haddad, MD*[1,6,7]

Author Affiliations:

[1]Department of Pediatrics, School of Medicine, University of California, San Diego, La Jolla, CA, USA.

[2]Department of Bioengineering, University of California, San Diego, La Jolla, CA, USA.

[3]Department of Cellular & Molecular Medicine, School of Medicine, University of California, San Diego, La Jolla, CA, USA.

[4]Department of Nanoengineering, University of California, San Diego, La Jolla, CA, USA.

[5]Department of Computer Science & Engineering, University of California, San Diego, La Jolla, CA, USA.

[6]Department of Neurosciences, School of Medicine, University of California, San Diego, La Jolla, CA, USA.

[7]Rady Children's Hospital, San Diego, CA, USA.

[†]Co-Second Authors
*Corresponding Author

Figure S1

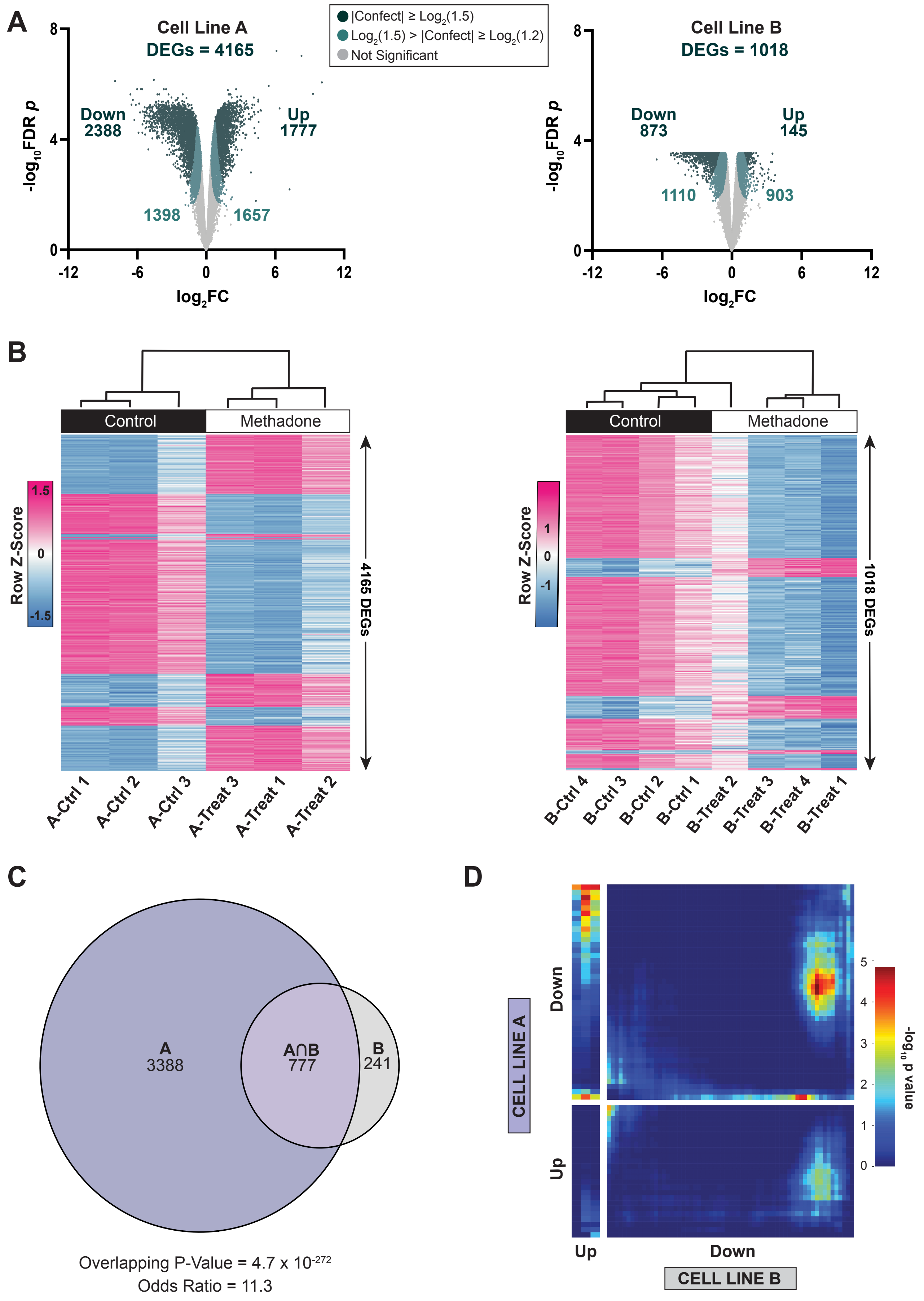

A
Cell Line A
DEGs = 4165
|Confect| ≥ Log2(1.5)
Log2(1.5) > |Confect| ≥ Log2(1.2)
Not Significant
Down
2388
Up
1777
1398
1657
-log10FDR p
log2FC
Cell Line B
DEGs = 1018
Down
873
Up
145
1110
903
B
Control
Methadone
Row Z-Score
4165 DEGs
A-Ctrl 1
A-Ctrl 2
A-Ctrl 3
A-Treat 3
A-Treat 1
A-Treat 2
1018 DEGs
B-Ctrl 4
B-Ctrl 3
B-Ctrl 2
B-Ctrl 1
B-Treat 2
B-Treat 3
B-Treat 4
B-Treat 1
C
A
3388
A∩B
777
B
241
Overlapping P-Value = 4.7 x 10-272
Odds Ratio = 11.3
D
CELL LINE A
Down
Up
CELL LINE B
-log10 p value

Figure S1. Robust transcriptional response of individual cell lines to chronic methadone treatment. (A) RNA-seq volcano plots of expressed genes in cell lines A and B, distinguished by their confident effect size 'confect' score cutoffs. DEGs were genes with $|\text{Confect}| \geq \log_2(1.5)$ and FDR < 0.05, following adjustment for cell-line differences in *limma* (Line A DEGs = 4165, Line B DEGs = 1018). For each gene, $\log_2$ (Fold Change) effect size values and Benjamini-Hochberg adjusted absolute $\log_{10}$ p-values, obtained using the Treat method in *limma*, are shown. **(B)** Heat maps depicting the sample-level expression of DEGs in cell lines A and B, respectively. Expression values are represented as z-scores. **(C)** Intersection of DEGs between cell lines A and B. Fisher's exact p-value and odds ratio demonstrating significance of overlap and strength of association, respectively, between DEG lists. **(D)** Rank-rank hypergeometric overlap heatmap depicting a high degree overlap between downregulated DEGs in cell lines A and B.

Figure S2

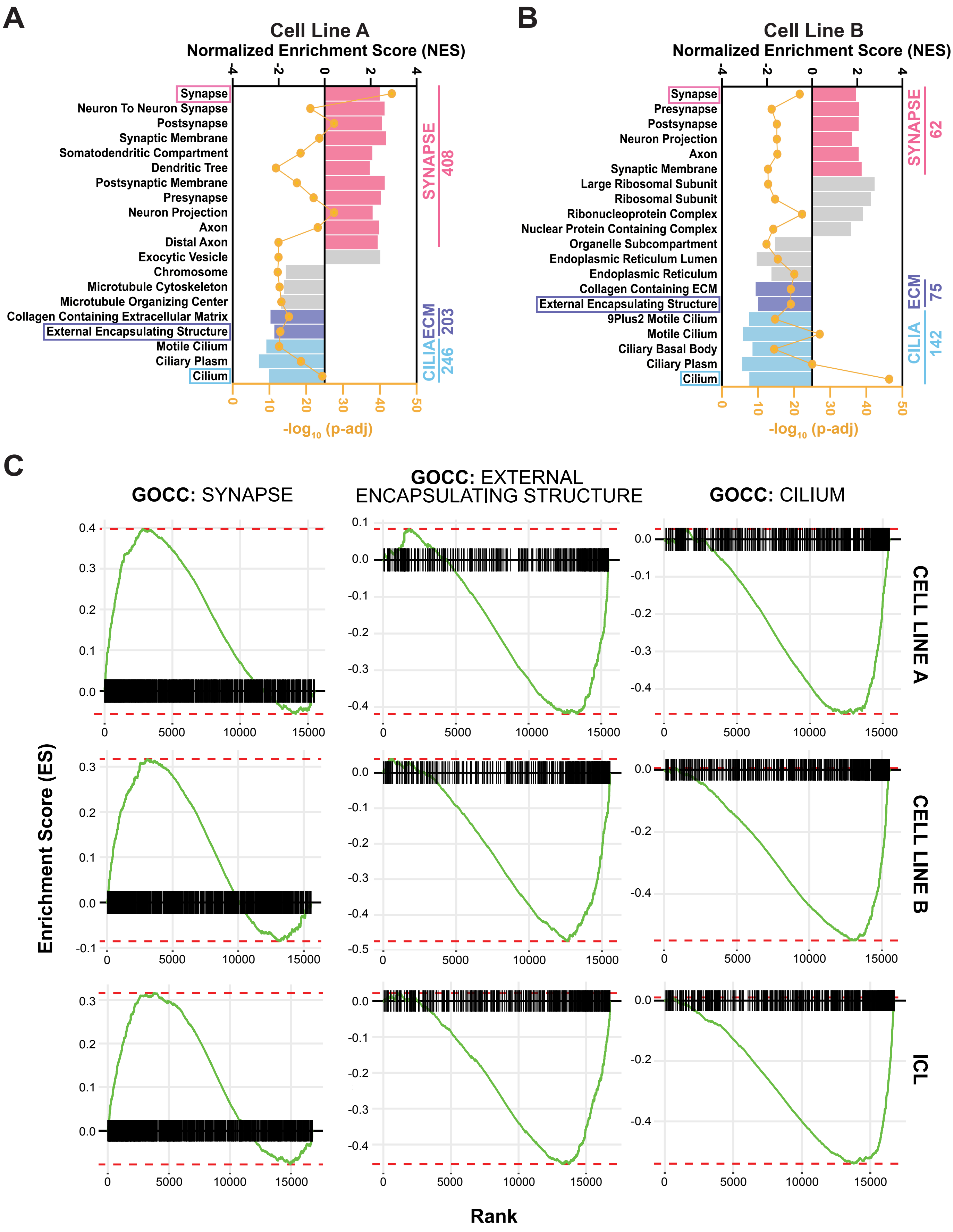
A
Cell Line A
Normalized Enrichment Score (NES)
Synapse
Neuron To Neuron Synapse
Postsynapse
Synaptic Membrane
Somatodendritic Compartment
Dendritic Tree
Postsynaptic Membrane
Presynapse
Neuron Projection
Axon
Distal Axon
Exocytic Vesicle
Chromosome
Microtubule Cytoskeleton
Microtubule Organizing Center
Collagen Containing Extracellular Matrix
External Encapsulating Structure
Motile Cilium
Ciliary Plasm
Cilium
SYNAPSE 408
ECM 203
CILIA 246
-log10 (p-adj)
B
Cell Line B
Normalized Enrichment Score (NES)
Synapse
Presynapse
Postsynapse
Neuron Projection
Axon
Synaptic Membrane
Large Ribosomal Subunit
Ribosomal Subunit
Ribonucleoprotein Complex
Nuclear Protein Containing Complex
Organelle Subcompartment
Endoplasmic Reticulum Lumen
Endoplasmic Reticulum
Collagen Containing ECM
External Encapsulating Structure
9Plus2 Motile Cilium
Motile Cilium
Ciliary Basal Body
Ciliary Plasm
Cilium
SYNAPSE 62
ECM 75
CILIA 142
-log10 (p-adj)
C
GOCC: SYNAPSE
GOCC: EXTERNAL ENCAPSULATING STRUCTURE
GOCC: CILIUM
CELL LINE A
CELL LINE B
ICL
Enrichment Score (ES)
Rank

Figure S2. DEGs in both cell lines are enriched for synaptic, ECM, and ciliary gene expression signatures. (A-B) Top 20 *fgsea* enriched GOCC gene sets in both cell lines based on absolute $\log_{10}$ FDR-adjusted p values. Gene sets were ranked according to the direction (positive or negative) of their normalized enrichment scores (NES) and grouped by cellular component. Values to the right of each graph indicate the number of DEGs associated with each of the top 3 enriched GO-CC gene sets (synapse, external encapsulating structure (ECM), and cilium). **(C)** *fgsea* enrichment plots for the top 3 enriched GOCC terms in each individual cell line and following adjustment for cell line differences (ICL, independent of cell line). Green lines represent the running enrichment score (ES) for each GO term as the analysis proceeds through the ranked list of expressed genes (black bars) in each dataset, peaking at the final ES score for that term.

Figure S3

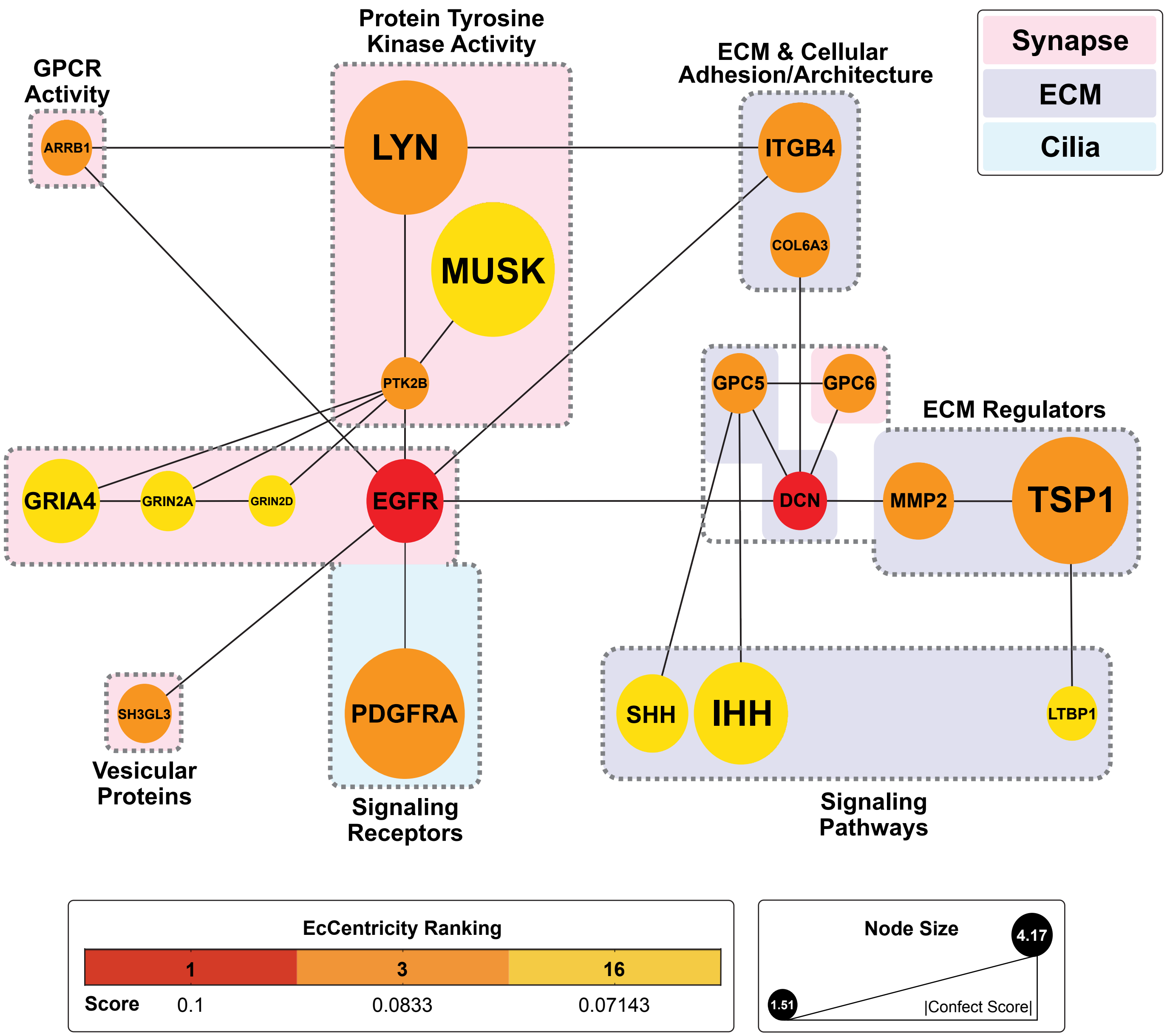

Protein Tyrosine Kinase Activity
GPCR Activity
ECM & Cellular Adhesion/Architecture
Synapse
ECM
Cilia
ARRB1
LYN
ITGB4
MUSK
COL6A3
PTK2B
GPC5
GPC6
ECM Regulators
GRIA4
GRIN2A
GRIN2D
EGFR
DCN
MMP2
TSP1
SH3GL3
PDGFRA
SHH
IHH
LTBP1
Vesicular Proteins
Signaling Receptors
Signaling Pathways
EcCentricity Ranking
1
3
16
Score
0.1
0.0833
0.07143
Node Size
4.17
1.51
|Confect Score|

Figure S3. Proteins encoded by synaptic and extra-synaptic hub DEGs comprise a central regulatory network. Predicted physical interactions between the top 20 hubs in a network of proteins encoded by co-expressed synaptic and extra-synaptic DEGs. The color of each node represents its centrality ranking according to the EcCentricity scores generated in *CytoHubba.* Both rankings and scores are shown in the legend below the network. Node sizes reflect the relative differential expression of each DEG based on absolute confect scores. Proteins are highlighted in purple, blue, or pink based on their association with synapses, the ECM, or cilia according to the GOCC database, and are clustered into broad functional groups (gray dotted lines).

Figure S4

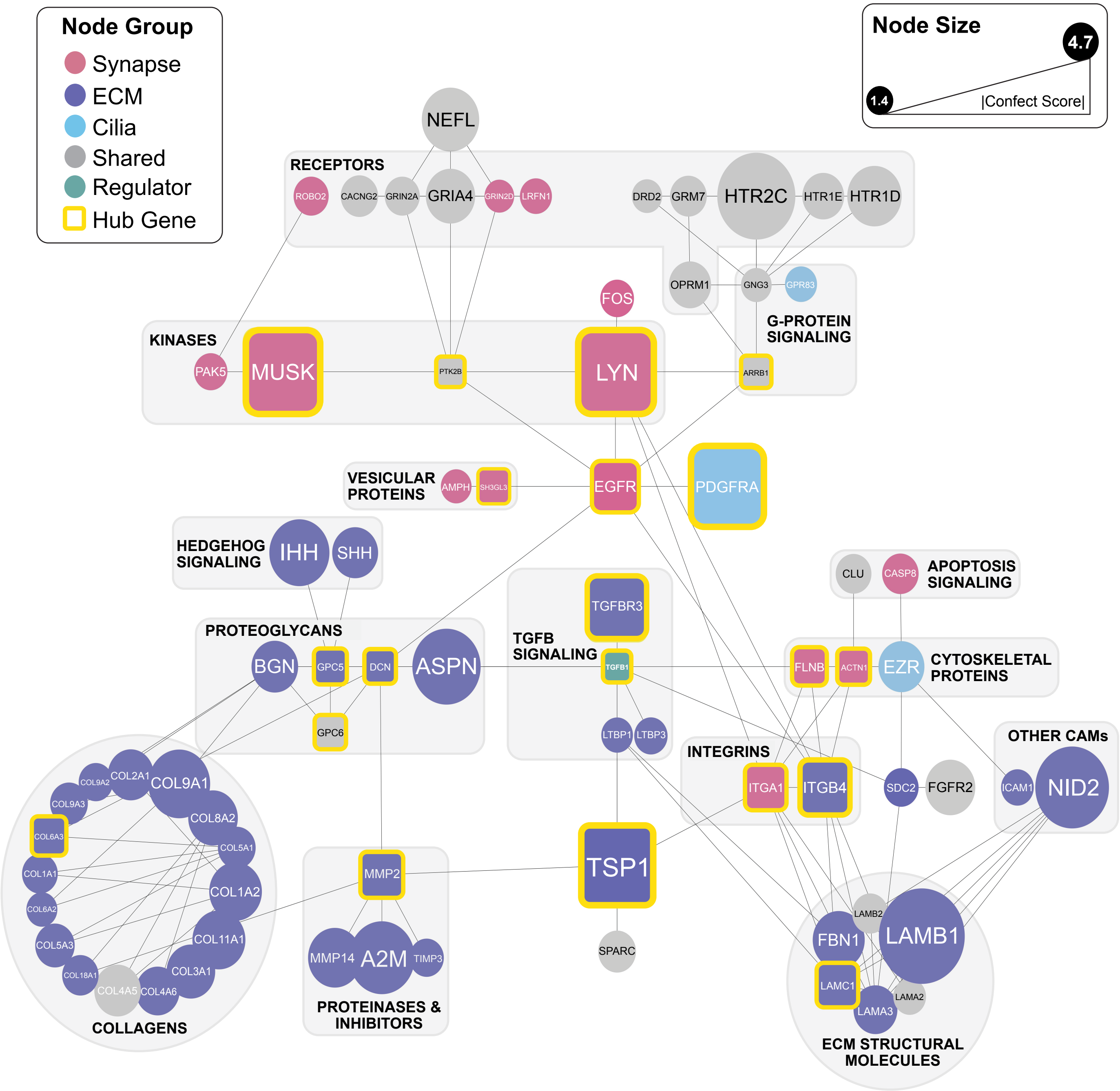
Node Group
Synapse
ECM
Cilia
Shared
Regulator
Hub Gene
Node Size
4.7
1.4
|Confect Score|
NEFL
RECEPTORS
ROBO2
CACNG2
GRIN2A
GRIA4
GRIN2D
LRFN1
DRD2
GRM7
HTR2C
HTR1E
HTR1D
OPRM1
GNG3
GPR83
G-PROTEIN SIGNALING
FOS
KINASES
PAK5
MUSK
PTK2B
LYN
ARRB1
VESICULAR PROTEINS
AMPH
SH3GL3
EGFR
PDGFRA
HEDGEHOG SIGNALING
IHH
SHH
APOPTOSIS SIGNALING
CLU
CASP8
PROTEOGLYCANS
BGN
GPC5
DCN
ASPN
GPC6
TGFB SIGNALING
TGFBR3
TGFB1
LTBP1
LTBP3
FLNB
ACTN1
EZR
CYTOSKELETAL PROTEINS
INTEGRINS
ITGA1
ITGB4
SDC2
FGFR2
OTHER CAMs
ICAM1
NID2
COL2A1
COL9A1
COL9A2
COL9A3
COL6A3
COL8A2
COL5A1
COL1A1
COL1A2
COL6A2
COL5A3
COL11A1
COL18A1
COL3A1
COL4A5
COL4A6
COLLAGENS
MMP2
MMP14
A2M
TIMP3
PROTEINASES & INHIBITORS
TSP1
SPARC
LAMB2
FBN1
LAMB1
LAMC1
LAMA2
LAMA3
ECM STRUCTURAL MOLECULES

Figure S4. Synaptic and extra-synaptic protein-protein interaction network including the top upstream regulator TGFB1. Proteins encoded by the synapse (pink), ECM (purple), and cilia (blue) associated DEGs belonging to the first two co-expression modules are shown, as well as the top upstream regulator TGFB1 (green). Nodes in grey represent genes belonging to more than one cellular component category. Node sizes reflect the relative magnitude of absolute confect scores, while edges indicate predicted physical interactions in the brain according to the STRING database. The top EcCentricity hubs identified by *CytoHubba* are emphasized with square boxes and yellow borders. Major known functional groups are shown in labeled grey boxes.

Table S1. Quality control information for each sequenced sample. RNA Integrity Numbers (RIN) and the total number of paired end reads are provided for each sequenced sample. The total reads for each sample are the sum of their forward (read 1) and reverse (read 2) reads.

	Sample ID	Treatment	RIN	Total Reads[a]
**Cell Line A**	A-Ctrl 1	Control	9.2	106,055,648
	A-Ctrl 2	Control	8.4	141,760,854
	A-Ctrl 3	Control	8.9	122,330,614
	A-Treat 1	Methadone	8.8	97,979,038
	A-Treat 2	Methadone	8.2	103,402,100
	A-Treat 3	Methadone	8.4	122,481,702
**Cell Line B**	B-Ctrl 1	Control	9.0	123,832,834
	B-Ctrl 2	Control	9.0	103,326,776
	B-Ctrl 3	Control	8.1	98,315,580
	B-Ctrl 4	Control	9.3	122,938,118
	B-Treat 1	Methadone	8.2	95,968,368
	B-Treat 2	Methadone	9.0	129,797,862
	B-Treat 3	Methadone	8.6	95,841,676
	B-Treat 4	Methadone	7.2	138,367,934

Table S2. Quantification of DEGs in each ECM molecular function category. The number of ECM-associated DEGs belonging to each molecular function category visualized in Figure 3B.

MatrisomeDB Division	MatrisomeDB Category	Molecule Type	Number of DEGs
**Core ECM**	**ECM Glycoproteins**	ECM Assembly Proteins	5
		Growth Factor Receptor/Binding Protein	5
		Laminin	5
		Other Cell Adhesion Molecules	4
		CCN Family Proteins	3
		Fibulin	3
		Integrin Signaling/Signal Transduction	2
		Nervous System Enriched ECM Proteins	2
		Collagen-Like/ Related	1
		Fibrillin	1
		Integrin	1
		Nidogen	1
		Protease Regulation	1
		Thrombospondin	1
	**Collagens**	Fibril Forming	7
		Fibril Associated	5
		Network-Forming	3
		Beaded Filament	2
	**Proteoglycans**	Extracellular Proteoglycan/SLRP	6
		Cell-Surface Proteoglycans	3
		Hyaluronan & Proteoglycan Link Protein	1
		Basement Membrane Proteoglycan	1
**Matrisome Associated**	**ECM Regulators**	Protease/Proteinase	16
		ECM Crosslinking/Remodeling Enzymes	8
		Protease/Proteinase Inhibitor	7
	**ECM Affiliated Proteins**	Annexins	6
		Nervous System Enriched ECM Proteins	4
		Other Basement Membrane Components	3
		Immune/Inflammatory Response	3
		Growth Factor Receptor/Binding Protein	2
		Growth Factor	1
		BMP Signaling	1
		Galectin	1
	**Secreted Factors**	WNT Signaling	4
		Growth Factor Signaling	2
		Angiopoietin Signaling	2
		BMP Signaling	2
		Hedgehog Signaling	2
		Annexin Signaling	1
		Apoptosis Signaling	1

Table S3. Top 10 enriched GOCC categories for co-expression modules 1 and 2. *fGSEA* using the GOCC database yielded the enrichment of synaptic, ciliary, and ECM associated transcriptional signatures among all genes in the top 2 co-expression modules. This data was used to generate the Sankey plot in Figure 4B.

	Pathway	$-\log_{10}$ (p-adj)	NES
**Module 1**	Cilium	34.185	-3.279
	Motile Cilium	21.080	-3.391
	Ciliary Plasm	14.217	-3.129
	Ciliary Basal Body	13.855	-3.073
	Axon	13.370	2.654
	Synapse	12.314	2.212
	Neuron Projection	12.179	2.210
	Collagen Containing Extracellular Matrix	11.417	-2.666
	External Encapsulating Structure	10.678	-2.471
	Postsynapse	9.578	2.463
**Module 2**	Synapse	10.293	2.253
	Neuron Projection	7.349	2.079
	Mitochondrial Matrix	7.349	-2.583
	Postsynapse	6.146	2.227
	Somatodendritic Compartment	6.146	2.152
	Neuron To Neuron Synapse	5.668	2.335
	Endoplasmic Reticulum Lumen	5.534	-2.439
	Presynapse	5.509	2.186
	Collagen Containing Extracellular Matrix	4.578	-2.278
	Dendritic Tree	4.470	2.065

Table S4. Networks of highly interconnected nodes associated with ECM structure and regulation. Highly interconnected sets of nodes identified in the synaptic-extra-synaptic network including the upstream regulator TGFB1 (Figure S4) using *MCODE*. For each cluster, the number of nodes and edges are provided, including a score calculated by multiplying the density of the cluster by the number of nodes.

Cluster	Score	Nodes	Edges	Node IDs
**1**	4.50	5	18	COL9A3, COL2A1, COL9A1, BGN, COL9A2
**2**	3.33	4	10	COL5A3, COL1A2, COL5A1, COL1A1
**3**	2.80	6	14	TGFB1, DCN, SPARC, GPC6, TSP1, GPC5

DATA FILE SUMMARY

Data File S1. *REVIGO* tree maps generated for DEGs as input for *CirGO* visualization. Each sheet contains a list of the enriched non-redundant GO-MF terms associated with the synaptic, ECM, and ciliary DEGs, respectively. Terms without a representative "parent" were assigned one based on the highest significant term in the GO hierarchy. This data was then used to generate the *CirGO* plots seen in Figures 2 and 3.

Data File S2. Molecular Functions of ECM and Synaptic DEGs. (Sheet 1-2) Categorization of synaptic DEGs (n = 166) and ECM DEGs (n = 129), the latter according to *Matrisome DB*. All genes grouped according to *OMIM, GeneCards,* and *NCBI Gene* databases**. (Sheet 3)** MCPs identified through published reviews. MCPs identified as classically found in the developing brain (Classical), identified via expansions of the MCP definition (Expanded), or proposed but are still under debate (Debated).

Data File S3. *IPA* Upstream Regulator Analysis output. Unfiltered output of all upstream regulators identified by Qiagen *IPA* for synaptic, ECM, and ciliary DEGs in co-expression Modules 1 and 2. Filtered list excluding exogenous toxins, chemicals, and drugs is also included.
